# An Image Reconstruction Algorithm for Electrical Capacitance Tomography Based on Robust Principle Component Analysis

**DOI:** 10.3390/s130202076

**Published:** 2013-02-05

**Authors:** Jing Lei, Shi Liu, Xueyao Wang, Qibin Liu

**Affiliations:** 1 Key Laboratory of Condition Monitoring and Control for Power Plant Equipment, Ministry of Education, North China Electric Power University, Beijing 102206, China; E-Mail: liushi_ncepu@yahoo.com.cn; 2 Institute of Engineering Thermophysics, Chinese Academy of Sciences, Beijing 100190, China; E-Mails: wangxueyao1982@126.com (X.W.); qibinliu@gmail.com (Q.L.)

**Keywords:** electrical capacitance tomography, capacitance sensor, image reconstruction, inverse problem, robust principle component analysis, forward-backward splitting technique, alternating direction iteration optimization method

## Abstract

Electrical capacitance tomography (ECT) attempts to reconstruct the permittivity distribution of the cross-section of measurement objects from the capacitance measurement data, in which reconstruction algorithms play a crucial role in real applications. Based on the robust principal component analysis (RPCA) method, a dynamic reconstruction model that utilizes the multiple measurement vectors is presented in this paper, in which the evolution process of a dynamic object is considered as a sequence of images with different temporal sparse deviations from a common background. An objective functional that simultaneously considers the temporal constraint and the spatial constraint is proposed, where the images are reconstructed by a batching pattern. An iteration scheme that integrates the advantages of the alternating direction iteration optimization (ADIO) method and the forward-backward splitting (FBS) technique is developed for solving the proposed objective functional. Numerical simulations are implemented to validate the feasibility of the proposed algorithm.

## 1. Introduction

Acquiring the spatial distribution information of materials is vital for improving the system efficiency and reducing pollution emission in chemical reactors or multiphase flow units. ECT is a noninvasive imaging technique, which is used to acquire spatial distribution information from inaccessible objects in order to monitor industrial processes. Owing to its distinct advantages such as the non-intrusive sensing, radiation-free nature, high temporal resolution, affordable measuring device and easy implementation, ECT is proven to be useful in industrial process monitoring, multiphase flow measurements, the visualization of combustion flames in porous media and the identification of two-phase flow patterns [1–10].

ECT technology attempts to reconstruct the permittivity distribution of the cross-section via an appropriate reconstruction algorithm from the capacitance measurement data, where reconstructing high-quality images plays a crucial role in real applications. Due to the ill-posed nature of the inverse problem, the ‘soft-field’ effect and the underdetermined problem in ECT image reconstruction, achieving high-accuracy reconstruction of a dynamic object is challenging. The key issue for improving the reconstruction quality has attracted intensive attention, and thus various algorithms, which can be approximately divided into two categories, static and dynamic reconstruction algorithms, had been developed for ECT image reconstruction. Common static reconstruction algorithms include the linear back-projection (LBP) method [11], the Tikhonov regularization method [12], the Landweber iteration algorithm [13–15], the offline iteration and online reconstruction (OIOR) technique [16], the truncated singular value decomposition (TSVD) method [17], the algebraic reconstruction technique (ART) [17], the simultaneous iterative reconstruction technique (SIRT) [17], the genetic algorithm (GA) [18], the generalized vector sampled pattern matching (GVSPM) method [19], the generalized Tikhonov regularization methods [20–23], the simulated annealing (SA) algorithm [24], the neural network algorithm [25], the level set method [26,27]. Detailed discussions about the numerical performance of other reconstruction algorithms can be found in [17,28].

The above-mentioned algorithms have played an important role in promoting the development of ECT technology and found numerous successful applications. It is worth mentioning that static reconstruction algorithms are often used to image a dynamic object [4,8]. However, these approaches exploit only the spatial relationship of the objects of interest, without using any temporal dynamics of the underlying process, which are not optimal for reconstructing a dynamic object unless the inversion solution is temporally uncorrelated. ECT measurement tasks often involve time-varying objects, and will be more applicable to image a dynamic object using a dynamic reconstruction algorithm that considers the temporal correlations of a dynamic object. In the field of ECT image reconstruction, dynamic reconstruction algorithms do not attract enough attention at present. Fortunately, several algorithms, such as the particle filter (PF) technique [29], the Kalman filter (KF) method [30] and the four-dimensional imaging algorithm [31], had been proposed for tackling the dynamic reconstruction tasks. Overall, the investigations of the dynamic reconstruction algorithms in the field of ECT are far from perfect, and finding an efficient dynamic reconstruction algorithm remains a critical issue.

Based on the RPCA method, a dynamic reconstruction model that utilizes the multiple measurement vectors is presented in this paper, where the evolution process of a dynamic object is regarded as a sequence of 2-D images with different temporal sparse deviations from a common background. An objective functional that simultaneously considers the temporal constraint and the spatial constraint is proposed, in which the images are reconstructed in a batching pattern. An iteration scheme that integrates the merits of the ADIO method and the FBS technique is developed for solving the established objective functional. Numerical simulations are implemented to validate the feasibility of the proposed algorithm.

The rest of this paper is organized as follows: based on the RPCA method, a reconstruction model that utilizes the multiple measurement vectors is proposed in Section 2. The original image reconstruction model is formulated into an optimization problem, and a new objective functional is established in Section 3. In Section 4, an iteration scheme that integrates the advantages of the ADIO method and the FBS algorithm is developed for solving the proposed objective functional. In Section 5, numerical simulations are implemented to evaluate the feasibility of the proposed algorithm, and a concise discussion on the numerical results is provided. Finally, conclusions are presented in Section 6.

## 2. Model Representation

### 2.1. Static Reconstruction Model

The ECT image reconstruction process involves two key phases: the forward problem and the inverse problem. The forward problem solves the capacitance values from a given permittivity distribution. It is worth mentioning that the forward problem is a well-posed problem, and it can be easily solved by numerical methods such as the finite element method or the finite difference method. The relationship between capacitance and the permittivity distribution can be formulated by [17]:

(1) $$C=\frac{Q}{V}=-\frac{1}{V}\iint_{\Gamma }\varepsilon (x,y)\nabla \phi (x,y)\text{d}\Gamma$$

where *Q* is the electric charge; *V* represents the potential difference between two electrodes forming the capacitance; *ε* (*x*, *y*) and *ϕ* (*x*, *y*) indicate the permittivity and electrical potential distributions, respectively; Г stands for the electrode surface.

The inverse problem attempts to estimate the permittivity distribution from the given capacitance data. In real applications, the static linearization image reconstruction model can be simplified as [17]:

(2) SG=C+r

where ***G*** is an *n*×1 dimensional vector standing for the normalized permittivity distributions; ***C*** represents an *m*×1 dimensional vector indicating the normalized capacitance values; ***r*** is an *m*×1 dimensional vector representing the capacitance measurement noises; ***S*** stands for a matrix of dimension *m*×*n*, and it is called as the sensitivity matrix in the field of ECT image reconstruction, which can be formulated by [32,33]:

(3) $${\text{S}}_{i,j}(x,y)=-\int_{p(x,y)}\frac{E_{i}(x,y)}{V_{i}}\cdot \frac{E_{j}(x,y)}{V_{j}}\text{d}x\text{d}y$$

where ***S****_i_*,*_j_* (*x*, *y*) defines the sensitivity between the *i*th electrode and the *j*th electrode at *p*(*x*, *y*); *E_i_*(*x*, *y*)stands for the electric field distribution inside the sensing domain when the *i*th electrode is activated as an excitation electrode by applying a voltage *V_i_* to it.

### 2.2. Multiple Measurement Vectors Dynamic Reconstruction Model

Equation (2) only considers the instantaneous measurement information, and uses single measurement data to implement image reconstruction without any considerations of the temporal dynamics of the underlying process, which is not optimal for reconstructing a dynamic object. It is well known that ECT reconstruction objects are often in a dynamic evolution process, and the measurement results at different time instants have a close correlation [4]. Therefore, considering such information may be important for imaging a dynamic object. In this paper, we propose a multiple measurement vectors dynamic reconstruction model, which can be formulated as:

(4) SX+U=Y+N

where ***U*** is an *m×t* dimensional matrix representing the model distortions derived from the facts [17,23,34], such as: (1) the simplification of a true physical process, (2) the linearization approximation distortions of the reconstruction model, and (3) physically implementing an ECT sensor; *t* >1 defines the number of measurements or the measurement time window; ***Y*** stands for an *m×t* dimensional matrix indicating the capacitance measurement data; ***X*** represents an *n×t* dimensional matrix, and each column of ***X*** stands for the permittivity distributions at the measurement time window *t*; ***N*** is an *m×t* dimensional matrix standing for the capacitance measurement noises.

In the static reconstruction model, the solution merely reflects an instantaneous measurement without any considerations of temporal dynamics of the underlying process, whereas in the case of the dynamic reconstruction model it reflects a sequence of temporally successive measurement, such that the temporal correlations of a dynamic object of interest should be imposed. In other word, the dependence of the capacitance measurement on the evolution of the permittivity distribution is explicitly considered in the dynamical reconstruction model. If the evolution of the permittivity distribution does not follow any dynamics, the dynamical reconstruction model reduces to the static reconstruction model. Obviously, the static reconstruction model is a special case of the dynamic reconstruction model.

The PCA method is an efficient data processing technique, which have enjoyed wide popularity in various fields. Unfortunately, the performances of the PCA technique suffer from the outliers in the data matrix, and thus different approaches had been developed for improving the PCA method. The RPCA method provides a new insight for modern data analysis approaches, which alleviates the deficiencies of the PCA method by applying the ℓ_1_-regularization and the nuclear norm on the matrix entries. Therefore, the RPCA method is robust to grossly corrupted observations of the underlying low-rank matrix. In a word, the RPCA method tries to recover principal component ***A*** (modeled by a low-rank matrix) from data matrix ***D*** with outliers ***E*** (modeled by a sparse matrix), which can be formulated into the following minimization problem under the certain conditions [35,36]:

(5) $$(\text{A},\text{E})=\min\left\{{\left\|\text{A}\right\|}_{*}+\lambda {\left\|\text{E}\right\|}_{1}\right\}\;\text{subject to}\;\text{D}=\text{A}+\text{E}$$

where, ‖·‖_*_ defines the nuclear norm for a matrix, and it can be specified as ${\left\|\text{A}\right\|}_{*}=\sum_{k}\sigma_{k}$, and *σ_k_* are the singular values of matrix ***A***; ‖· ‖_1_ represents the ℓ_1_-norm for a matrix, which can be defined as ${\left\|\text{E}\right\|}_{1}=\sum_{j}\left(\sum_{i}\left|{\text{E}}_{i,j}\right|\right)$; *λ* > 0 is a regularization parameter. It is worth mentioning that term **‖*E*‖**_1_ is introduced to satisfy the sparsity assumption of matrix ***E***.

Studies indicate that the evolution process of a dynamic object can be regarded as a sequence of images with different temporal sparse deviations from a common background. Motivated by this observation, the following low-rank and sparse decomposition of ***X*** can be obtained [37]:

(6) $$\text{X}={\text{X}}_{1}+{\text{X}}_{2}$$

where ***X***_1_ is the low-rank matrix component for modeling the ‘background components’ of ***X***. It is worth mentioning that ***X***_1_ is assumed to resemble each other rather than to be constant in time, which can be described as a low-rank matrix in mathematics under the RPCA framework [37]. On the other hand, ***X***_2_ is the sparse matrix component for modeling the sparse deviation from the background ***X***_1_. Submitting Equation (6) to Equation (4) yields the following expression:

(7) $$\text{S}({\text{X}}_{1}+{\text{X}}_{2})+\text{U}=\text{Y}+\text{N}$$

## 3. Design of the Objective Functional

Equation (7) is a matrix-based reconstruction model, and it includes three unknown matrix variables. Therefore, it is hard to directly solve the equation. ECT image reconstruction process is a typical ill-posed problem, methods that ensure the numerical stability while improving the quality of an inversion solution should be employed. The Tikhonov regularization technique is an efficient method for solving the ill-posed problems, which has enjoyed wide popularity in various fields. A Tikhonov regularization solution is essentially a result of balancing the accuracy and stability of an inversion solution. According to the Tikhonov regularization method and the optimization theory, Equation (7) can be reformulated as:

(8) $$\min\left\{\frac{1}{2}{\left\|\text{S}({\text{X}}_{1}+{\text{X}}_{2})+\text{U}-\text{Y}\right\|}_{F}^{2}+\alpha_{1}{\left\|{\text{X}}_{1}\right\|}_{*}+\alpha_{2}{\left\|{\text{X}}_{2}\right\|}_{1}+\alpha_{3}\Omega (\text{U})+\frac{\alpha_{4}}{2}{\left\|({\text{X}}_{1}+{\text{X}}_{2})\text{W}\right\|}_{F}^{2}\right\}$$

where operator ‖·‖*_F_* defines the Frobenius norm for a matrix; ${\left\|\text{S}({\text{X}}_{1}+{\text{X}}_{2})+\text{U}-\text{Y}\right\|}_{F}^{2}=\text{Tr}({\left(\text{S}({\text{X}}_{1}+{\text{X}}_{2})+\text{U}-\text{Y}\right)}^{T}(\text{S}({\text{X}}_{1}+{\text{X}}_{2})+\text{U}-\text{Y}))$, and Tr(·) represents the trace of a matrix; *α*_1_ > 0, *α*_2_ > 0, *α*_3_ > 0 and *α*_4_ > 0 are the regularization parameters; Ω represents a stabilizing functional; ${\left\|({\text{X}}_{1}+{\text{X}}_{2})\text{W}\right\|}_{F}^{2}=\text{Tr}({\left(({\text{X}}_{1}+{\text{X}}_{2})\text{W}\right)}^{T}(({\text{X}}_{1}+{\text{X}}_{2})\text{W}))$ stands for a temporal constraint, where ***W*** is defined as:

(9) $$\text{W}={\left[\begin{array}{llll} -1 & 0 & \cdots & 0\\ 1 & -1 & \cdots & 0\\ 0 & 1 & \cdots & 0\\ \vdots & \vdots & \cdots & \vdots \\ 0 & 0 & \cdots & -1\\ 0 & 0 & \cdots & 1 \end{array}\right]}_{t\times t-1}$$

Equation (8) can be called as a generalized Tikhonov functional, where ${\left\|\text{S}({\text{X}}_{1}+{\text{X}}_{2})+\text{U}-\text{Y}\right\|}_{F}^{2}$ measures the accuracy of an inversion solution, and ‖***X***_1_‖_*_, ‖***X***_2_‖_1_, Ω(***U***) and ${\left\|({\text{X}}_{1}+{\text{X}}_{2})\text{W}\right\|}_{F}^{2}$ achieve the numerical stability. Particularly, terms ‖***X***_1_‖_*_ and ‖***X***_2_‖_1_ are used to model the background component and the sparse deviation from the background of a dynamic process, respectively. It is worth mentioning that ${\left\|({\text{X}}_{1}+{\text{X}}_{2})\text{W}\right\|}_{F}^{2}$ can be considered as a temporal constraint, which is introduced to impose the temporal correlation of a dynamic object.

The design of function Ω is vital for Equation (8). For simplicity, function Ω is defined as:

(10) $$\Omega (\text{U})={\left\|\text{U}\right\|}_{1}$$

Following the discussions presented in previous sections, an objective functional for ECT image reconstruction can be specified by:

(11) $$\min\left\{\frac{1}{2}{\left\|\text{S}({\text{X}}_{1}+{\text{X}}_{2})+\text{U}-\text{Y}\right\|}_{F}^{2}+\alpha_{1}{\left\|{\text{X}}_{1}\right\|}_{*}+\alpha_{2}{\left\|{\text{X}}_{2}\right\|}_{1}+\alpha_{3}{\left\|\text{U}\right\|}_{1}+\frac{\alpha_{4}}{2}{\left\|({\text{X}}_{1}+{\text{X}}_{2})\text{W}\right\|}_{F}^{2}\right\}$$

Several desirable properties can be found from Equation (11):

1. In traditional reconstruction models, only single measurement data is used to independently implement image reconstruction; in Equation (11), however, multiple measurement vectors are employed to image a dynamic object and the images are reconstructed by a batching pattern, which differs from other vector-based reconstruction algorithms such as the ART method and the GVSPM algorithm.
2. In Equation (11), the image series are divided into two matrix-based components by the RPCA method, and the evolution process of a dynamic object is considered as a sequence of images with different temporal sparse deviations from a common background. It is worth mentioning that in real applications different constraints can be imposed on the two matrix-based components according to the prior information of a dynamic object, which will facilitate the improvement of the reconstruction quality.
3. In Equation (11), the temporal constraint and the spatial constraint are simultaneously considered. Particularly, the temporal constraint is introduced to impose the temporal correlations of a dynamic object. Furthermore, the nuclear norm and the ℓ_1_-norm are used to model the background component and the sparse component of a dynamic evolution process, respectively.
4. The measurement noises and the model approximation distortions are simultaneously considered in Equation (11), which is highly suitable for real applications. In particular, the accuracy measure of an inversion solution is considered under a measurement time window, which differs from single measurement vector-based methods.
5. ECT image reconstruction process is a typical ill-posed problem, methods that ensure the numerical stability while improving the quality of an inversion solution should be employed. In Equation (11), the Tikhonov regularization technique is introduced to ensure the numerical stability of an inversion solution.
6. The unknown variables in Equation (11) are three matrices, and the computational approaches and strategies will be distinctly different from other vector-based reconstruction algorithms such as the ART method and the GVSPM algorithm.

## 4. Solving of the Objective Functional

Developing an efficient algorithm to solve Equation (11) is crucial for real applications of the proposed dynamic reconstruction model. In this section, the ADIO method and the FBS technique are concisely introduced, and an iteration scheme that integrates the advantages of the both methods is developed for solving Equation (11).

### 4.1. Alternating Direction Iteration Optimization Method

Equation (11) includes three unknown matrix variables ***U***, ***X***_1_ and ***X***_2_, and directly solving the equation is challenging. In the ADIO method, different unknown variables can be alternately solved [38], and thus Equation (11) can be reformulated into the following three sub-problems:

(12) $${\text{U}}^{k+1}=\underset{\text{U}}{\min}\left\{\frac{1}{2}{\left\|\text{S}({\text{X}}_{1}^{k}+{\text{X}}_{2}^{k})+\text{U}-\text{Y}\right\|}_{F}^{2}+\alpha_{3}{\left\|\text{U}\right\|}_{1}\right\}$$

where ${\left\|\text{S}({\text{X}}_{1}^{k}+{\text{X}}_{2}^{k})+\text{U}-\text{Y}\right\|}_{F}^{2}=\text{Tr}({\left(\text{S}({\text{X}}_{1}^{k}+{\text{X}}_{2}^{k})+\text{U}-\text{Y}\right)}^{T}(\text{S}({\text{X}}_{1}^{k}+{\text{X}}_{2}^{k})+\text{U}-\text{Y}))$.

(13) $${\text{X}}_{1}^{k+1}=\underset{{\text{X}}_{1}}{\min}\left\{\frac{1}{2}{\left\|\text{S}({\text{X}}_{1}+{\text{X}}_{2}^{k})+{\text{U}}^{k+1}-\text{Y}\right\|}_{F}^{2}+\alpha_{1}{\left\|{\text{X}}_{1}\right\|}_{*}+\frac{\alpha_{4}}{2}{\left\|({\text{X}}_{1}+{\text{X}}_{2}^{k})\text{W}\right\|}_{F}^{2}\right\}$$

where ${\left\|\text{S}({\text{X}}_{1}+{\text{X}}_{2}^{k})+{\text{U}}^{k+1}-\text{Y}\right\|}_{F}^{2}=\text{Tr}({\left(\text{S}({\text{X}}_{1}+{\text{X}}_{2}^{k})+{\text{U}}^{k+1}-\text{Y}\right)}^{T}(\text{S}({\text{X}}_{1}+{\text{X}}_{2}^{k})+{\text{U}}^{k+1}-\text{Y}))$ and ${\left\|({\text{X}}_{1}+{\text{X}}_{2}^{k})\text{W}\right\|}_{F}^{2}=\text{Tr}({\left(({\text{X}}_{1}+{\text{X}}_{2}^{k})\text{W}\right)}^{T}(({\text{X}}_{1}+{\text{X}}_{2}^{k})\text{W}))$.

(14) $${\text{X}}_{2}^{k+1}=\underset{{\text{X}}_{2}}{\min}\left\{\frac{1}{2}{\left\|\text{S}({\text{X}}_{1}^{k+1}+{\text{X}}_{2})+{\text{U}}^{k+1}-\text{Y}\right\|}_{F}^{2}+\alpha_{2}{\left\|{\text{X}}_{2}\right\|}_{1}+\frac{\alpha_{4}}{2}{\left\|({\text{X}}_{1}^{k+1}+{\text{X}}_{2})\text{W}\right\|}_{F}^{2}\right\}$$

where ${\left\|\text{S}({\text{X}}_{1}^{k+1}+{\text{X}}_{2})+{\text{U}}^{k+1}-\text{Y}\right\|}_{F}^{2}=\text{Tr}({\left(\text{S}({\text{X}}_{1}^{k+1}+{\text{X}}_{2})+{\text{U}}^{k+1}-\text{Y}\right)}^{T}(\text{S}({\text{X}}_{1}^{k+1}+{\text{X}}_{2})+{\text{U}}^{k+1}-\text{Y}))$ and ${\left\|({\text{X}}_{1}^{k+1}+{\text{X}}_{2})\text{W}\right\|}_{F}^{2}=\text{Tr}({\left(({\text{X}}_{1}^{k+1}+{\text{X}}_{2})\text{W}\right)}^{T}(({\text{X}}_{1}^{k+1}+{\text{X}}_{2})\text{W}))$.

According to the above discussions, Equation (11) is divided into three sub-problems, in which Equation (12) can be solved by the shrinkage algorithm [39]. Obviously, solving Equations (13) and (14) plays a crucial role in real applications.

### 4.2. Forward-Backward Splitting Technique

The FBS technique is originally proposed for solving the following optimization problem [40–44]:

(15) $$\underset{\text{u}}{\min}\left\{\mu J(\text{u})+H(\text{u})\right\}$$

where *J* and *H* are the known functions.

According to the corresponding deductions, the resulting FBS algorithm can be formulated as:

(16) $${\text{u}}^{k+1}={\text{Prox}}_{\delta \mu J}({\text{u}}^{k}-\delta \partial H({\text{u}}^{k}))$$

where the proximal operator Prox*_δμJ_* (***ν***) is defined as:

(17) $$\underset{\text{u}}{\arg\min}\left\{\mu J(\text{u})+\frac{1}{2\delta }{\left\|\text{u}-\text{v}\right\|}^{2}\right\}$$

In the case of $H(\text{u})=\frac{1}{2}{\left\|\text{Au}-\text{f}\right\|}^{2}$, we can obtain ∂*H*(***u***) = ***A****^T^*(***Au*** − ***f***). Therefore, Equation (15) can be solved by the following two-step algorithm:

(18) $${\text{v}}^{k+1}={\text{u}}^{k}-\delta {\text{A}}^{T}({\text{Au}}^{k}-\text{f})$$

(19) $${\text{u}}^{k+1}=\underset{\text{u}}{\arg\min}\left\{\mu J(\text{u})+\frac{1}{2\delta }{\left\|\text{u}-{\text{v}}^{k+1}\right\|}^{2}\right\}$$

### 4.3. Proposed Iteration Scheme

Following the above discussions, an iteration scheme can be developed for solving Equation (11), which can be summarized as follows:

Step 1. Specify the algorithmic parameters and the initial values.
Step 2. Update variable ***U*** by solving Equation (12) using the shrinkage algorithm [39].
Step 3. Update variable ***X***_1_ by solving Equation (13) using the FBS algorithm and the singular value thresholding (SVT) technique [45].
Step 4. Update variable ***X***_2_ by solving Equation (14) using the FBS method.
Step 5. Loop to Step 2 until a predetermined iteration stopping criterion is satisfied.

Additionally, it can be known in advance that the inversion solution belongs to the range [Θ_1_, Θ_2_ ], therefore a projected operator is introduced to the iteration scheme:

(20) $${\text{X}}^{k+1}=\text{Project}\left\{{\text{X}}^{k+1}\right\}$$

where:

(21) $$\text{Project}\left[{\text{D}}_{i,j}\right]=\{\begin{array}{ll} \Theta_{1}, & \text{if}\;{\text{D}}_{i,j}<\Theta_{1}\\ f(x), & \text{if}\;\Theta_{1}\leq {\text{D}}_{i,j}\leq \Theta_{2}\\ \Theta_{2} & \text{if}\;{\text{D}}_{i,j}>\Theta_{2} \end{array}$$

## 5. Numerical Simulations and Discussions

**Figure 1.**
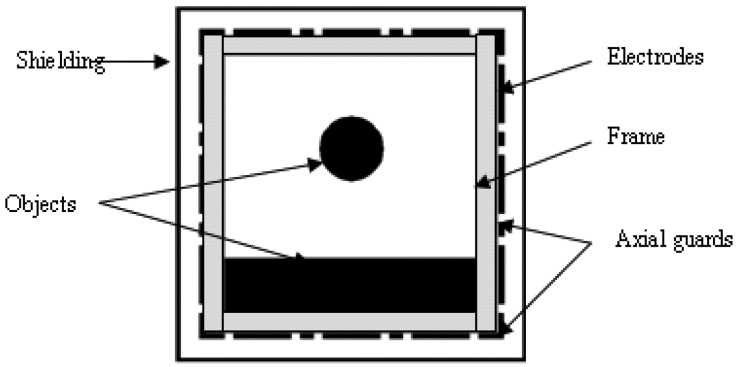
Layout of ECT sensor.

According to the above discussions, the proposed reconstruction technique can be concisely called as the multiple measurement vectors dynamic reconstruction (MMVDR) algorithm. In this section, dynamic reconstruction cases are implemented to evaluate the feasibility of the MMVDR algorithm, and the reconstruction quality is compared with the projected Landweber iteration (PLI) method. The initial values are computed by the standard Tikhonov regularization method. All algorithms are implemented using the MATLAB 7.0 software on a PC with a Pentium IV 2.4 G Hz CPU, and 4 G bytes memory. The image error is used to evaluate the reconstruction quality, which is defined as [17]:

(22) $$\eta =\frac{\left\|{\text{G}}_{\text{True}}-{\text{G}}_{\text{Estimated}}\right\|}{\left\|{\text{G}}_{\text{True}}\right\|}\times 100\%$$

where, ***G***_True_ and ***G***_Estimated_ represent the true and estimated permittivity distributions, respectively. A 12 electrodes square ECT sensor, which is present in Figure 1, is selected for simulations and an image is presented using 32 × 32 pixels.

Figure 1 illustrates a square ECT sensor, which contains an array of 12 electrodes around the frame. The sensor is enclosed by an earthed shielding to protect the sensor against the effects of external charged objects. The size of the frame is 80 × 80 mm, and the width of the frame is 6 mm. The length and width of the electrodes are 18 mm and 1 mm, respectively. The length and width of the axial guards are 4 mm and 1 mm, respectively. The size of shielding is 108 × 108 mm.

### 5.1. Case 1

**Figure 2.**
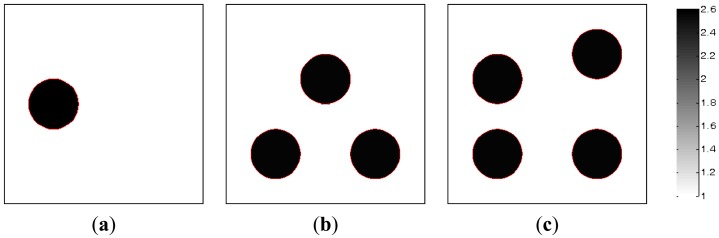
Dynamic reconstruction objects.

In the section, the image reconstructions with three measurement vectors (*t* = 3) are firstly implemented to evaluate the feasibility of the MMVDR algorithm and the reconstruction quality is compared with the PLI method. Subfigures a, b and c in Figure 2, where the black color stands for the high permittivity materials with a value of 2.6 and the white color represents the low permittivity materials with a value of 1.0, stand for the dynamic permittivity distributions of a dynamic object at time instants *t*, *t* + 1 and *t* +2. The diameters of the cylinders in Figure 2 are 20 mm.

**Figure 3.**
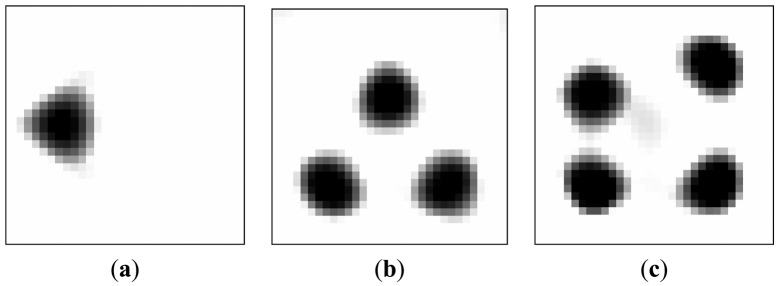
Reconstructed images by the PLI algorithm.

**Figure 4.**
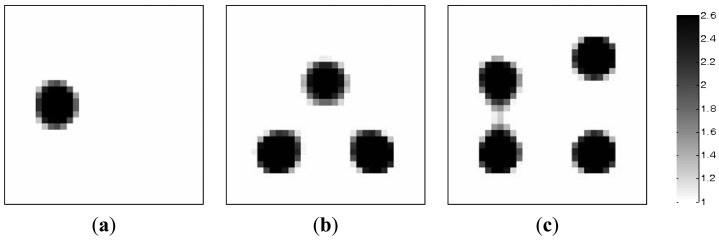
Reconstructed images by the MMVDR algorithm.

Table 1 lists the algorithmic parameters for the PLI method. In the MMVDR algorithm, *α*_1_ = 1.5, *α*_2_ = 0.05, *α*_3_ = 0.07, *α*_4_ = 0.001, and the number of iterations is 130. Figures 3 and 4 illustrate the images reconstructed by the PLI method and the MMVDR algorithm, respectively. The image errors are shown in Table 2.

The images reconstructed by the PLI method are presented in Figure 3. Numerical simulation results indicate that distinct advantages of the PLI method involve easy numerical implementation and low computational complexity and cost owing to the fact that only gradient information of the objective functional is used. However, the PLI fails to consider the temporal correlations of a dynamic object, and the improvement of the reconstruction quality is therefore restricted. For the cases simulated in this section, the quality of the images reconstructed by the PLI method is far from perfect and the distortions are relatively serious.

The images reconstructed by the MMVDR algorithm are illustrated in Figure 4. Numerical simulation results indicate that the MMVDR algorithm can ensure the numerical stability of an inversion solution owing to the fact that the Tikhonov regularization technique is used to stabilize the numerical solution. Particularly, the computational complexity and cost of the MMVDR algorithm are low, and the numerical implementation is easy. As can be expected, it can be seen from Figure 4 that owing to the fact that the temporal constraint and spatial constraint are simultaneously considered, the quality of the images reconstructed by the MMVDR algorithm is improved as compared to the PLI method, which indicates that the MMVDR algorithm is competent in solving ECT inverse problems.

Meanwhile, it can be found that in the MMVDR algorithm, the images are reconstructed by a batching pattern, and thus the temporal correlations of a dynamic object can be better considered, which differs from other vector-based reconstruction algorithms. In addition, it can be found that the MMVDR method is an iterative algorithm, and it is hard to achieve the online reconstruction presently. In the future, more investigations on improving reconstruction speed should be implemented.

The image errors are listed in Table 2. It can be found that the quality of the images reconstructed by the MMVDR algorithm is higher than with the PLI method, which confirms that the MMVDR algorithm is a promising candidate for solving ECT image reconstruction problems.

### 5.2. Case 2

In this section, the image reconstructions with four measurement vectors (*t* = 4) are implemented to further evaluate the feasibility of the MMVDR algorithm, and the reconstruction quality is compared with the PLI method.

**Figure 5.**
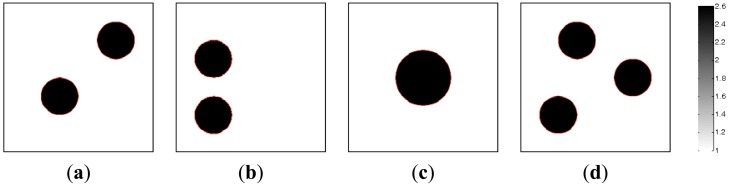
Dynamic reconstruction objects.

Figure 5 simulates the dynamic process of the objects of interest under different time instants. Subfigures a, b, c and d in Figure 5, where the black color stands for the high permittivity materials with a value of 2.6 and the white color represents the low permittivity materials with a value of 1.0, represent the dynamic permittivity distributions of a dynamic object at time instants *t*, *t* + 1, *t* + 2 and *t* + 3. The diameters of the cylinders in the subfigures a, b and d in Figure 5 are 20 mm, and the diameter of the cylinder in Figure 5(c) are 30 mm.

**Figure 6.**
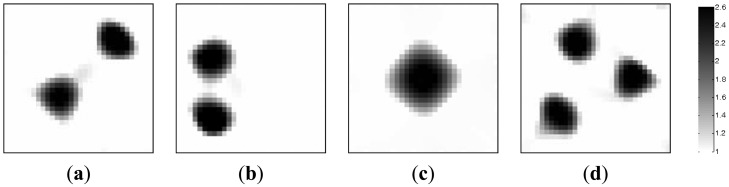
Reconstructed images by the PLI algorithm.

**Figure 7.**
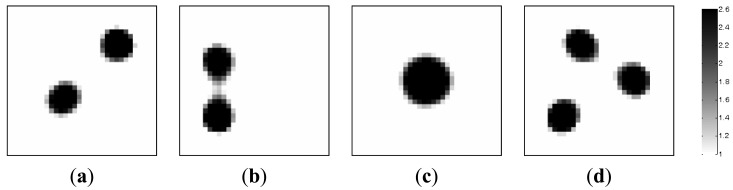
Reconstructed images by the MMVDR algorithm.

Algorithmic parameters for the PLI method are listed in Table 3. The algorithmic parameters for the MMVDR algorithm are the same as Section 5.1. The images reconstructed by the PLI method and the MMVDR algorithm are illustrated in Figures 6 and 7, respectively. Table 4 lists the image errors for the PLI algorithm and the MMVDR algorithm.

The images reconstructed by the PLI method are presented in Figure 6. It can be seen that for the dynamic reconstruction case simulated in this section, the quality of the images reconstructed by the PLI method is far from perfect and the distortions are relatively large.

Figure 7 illustrates the images reconstructed by the MMVDR algorithm. As can be expected, it can be seen from Figure 7 that the quality of the images reconstructed by the MMVDR algorithm is improved as compared to the PLI method. At the same time, it can be observed from Table 4 that for the case simulated in this section, the MMVDR algorithm gives the smallest image errors, which indicates that the MMVDR algorithm is successful in solving ECT inverse problems.

### 5.3. Case 3

In this section, the noise-contaminated capacitance data is used to evaluate the robustness of the MMVDR algorithm. In this case, two measurement vectors (*t* = 2) are used to implement the image reconstruction. The noise level is defined by [17]:

(23) $$\gamma =\frac{\left\|{\text{C}}_{\text{Contaminated}}-{\text{C}}_{\text{True}}\right\|}{\left\|{\text{C}}_{\text{True}}\right\|}\times 100\%$$

Where *γ* is the noise level; ***C***_True_ stands for the true capacitance data; ***C***_Contaminated_ is the noise-contaminated capacitance data; ***C***_Contraminated_ = ***C***_True_ + ***r***, where ***r*** = *σ*_1_ · *randn*; *σ*_1_ represents the standard deviation and *randn* stands for a Gaussian distribution random number with the mean of 0 and the standard deviation of 1, which can be achieved by the function ‘randn’ in the MATLAB software

**Figure 8.**
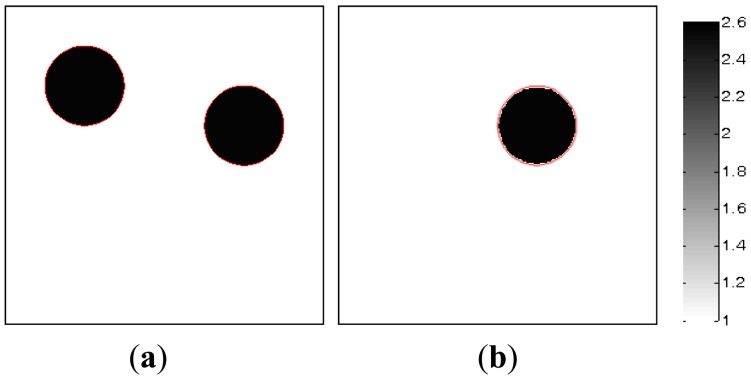
Dynamic reconstruction objects.

Figure 8 shows the dynamic process of the objects of interest, in which subfigures a and b represent the dynamic permittivity distributions of a dynamic object at time instants *t* and *t* + 1. In Figures 8(a) and b, the diameters of the cylinders are 20 mm, the permittivity of the cylinder is 2.6, and the permittivity of the rest of the reconstruction region is 1.0.

**Figure 9.**
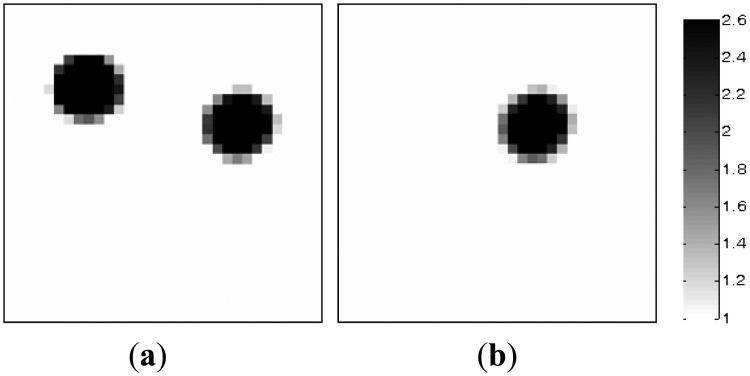
Reconstructed images by the MMVDR algorithm under the noise level of 0%.

**Figure 10.**
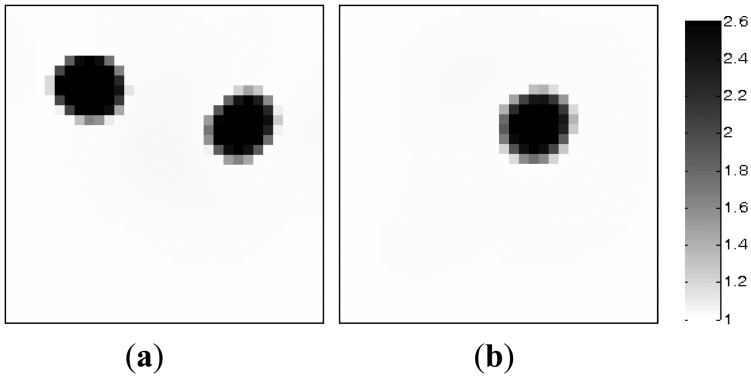
Reconstructed images by the MMVDR algorithm under the noise level of 9%.

**Figure 11.**
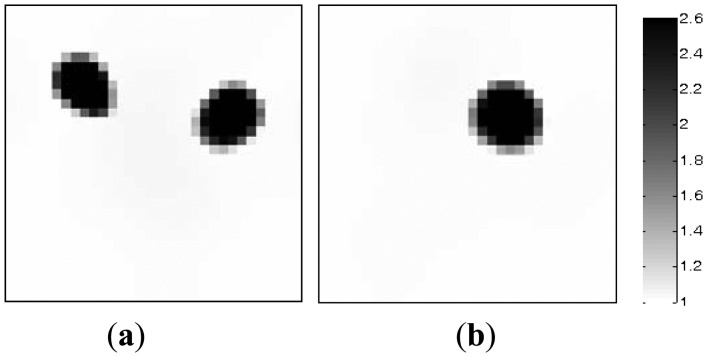
Reconstructed images by the MMVDR algorithm under the noise level of 24%.

**Figure 12.**
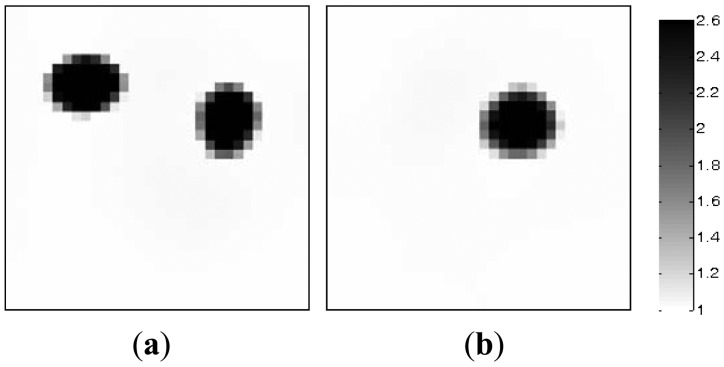
Reconstructed images by the MMVDR algorithm under the noise level of 33%.

In the MMVDR algorithm, the algorithmic parameters are the same as Section 5.1. Figures 9–12 show the images reconstructed by the MMVDR algorithm under the noise levels of 0, 9%, 24% and 33%. The image errors are listed in Table 7.

Figures 9–12 are the images reconstructed by the MMVDR algorithm under the noise levels of 0, 9%, 24% and 33%, respectively. As can be expected, it can be seen from Figures 9–12 that the MMVDR algorithm shows satisfactory robustness, and the quality of the images reconstructed under different noise levels is acceptable, which is highly desirable for real applications owing to the fact that the measurement noises are inevitable in practice. When the noise level is 33%, especially, the image errors for the dynamic reconstruction objects, Figure 8(a,b), are 13.81% and 10.20%, which indicates that the MMVDR algorithm is successful in treating with the measurement noises. Additionally, it can be seen from Figures 9–12 and Table 5 that the image errors increase with the increment of the noise levels, which indicates that the inaccuracy of the capacitance measurement data should be seriously tackled and this issue should be further studied in the future.

## 6. Conclusions

ECT is considered a promising visualization measurement technology, in which reconstructing high quality images is highly desirable for real applications. In this paper, based on the RPCA technique, a dynamic reconstruction model that utilizes the multiple measurement vectors is presented, in which the evolution process of a dynamic object is considered as a sequence of images with different temporal sparse deviations from a common background. An objective functional that simultaneously considers the temporal constraint and the spatial constraint is proposed, where the images are reconstructed in a batching pattern. An iteration scheme that integrates the advantages of the ADIO method and the FBS technique is developed for solving the proposed objective functional. Numerical simulation results indicate that the proposed algorithm can ensure a stable numerical solution. For the cases simulated in this paper, the quality of the images reconstructed by the proposed algorithm is improved, which indicates that the proposed algorithm is successful in solving ECT inverse problems. As a result, a promising algorithm is introduced for ECT image reconstruction.

Applications indicate that each algorithm may show different numerical performances to different reconstruction tasks. In practice, the selection of a reconstruction algorithm depends mainly on the measurement requirements and the prior information of a specific reconstruction task. Our work provides an alternative approach for solving ECT inverse problems, which needs to be validated by more cases in the future. At the same time, more investigations on the improvement of the reconstruction speed should be undertaken.

## Figures and Tables

**Table 1.** Algorithmic parameters for the PLI algorithm.

**Algorithmic Parameters**	Figure 3(a)	Figure 3(b)	Figure 3(c)
Relaxation factor	1	1	1
Number of iteration	480	401	432

**Table 2.** Image errors (%).

**Algorithms**	Figure 2(a)	Figure 2(b)	Figure 2(c)
PLI	12.61	18.03	18.82
MMVDR	9.08	13.92	15.11

**Table 3.** Algorithmic parameters for the PLI algorithm.

**Algorithmic Parameters**	Figure 6(a)	Figure 6(b)	Figure 6(c)	Figure 6(d)
Relaxation factor	1	1	1	1
Number of iteration	535	500	111	748

**Table 4.** Image errors (%).

**Algorithms**	Figure 5(a)	Figure 5(b)	Figure 5(c)	Figure 5(d)
PLI	15.77	15.09	15.22	18.39
MMVDR	10.37	11.55	6.68	13.40

**Table 5.** Image errors (%).

**Noise levels**	Figure 8(a)	Figure 8(b)
0	10.89	7.36
9%	11.03	7.87
24%	12.63	10.06
33%	13.81	10.20

## References

[1] Rimpilainen V., Heikkinen L.M., Vauhkonen M. (2012). Moisture distribution and hydrodynamics of wet granules during fluidized-bed drying characterized with volumetric electrical capacitance tomography. Chem. Eng. Sci..

[2] Rimpilainen V., Poutiainen S., Heikkinen L.M., Savolainen T., Vauhkonen M., Ketolainen J. (2011). Electrical capacitance tomography as a monitoring tool for high-shear mixing and granulation. Chem. Eng. Sci..

[3] Wang H.G., Yang W.Q. (2010). Measurement of fluidised bed dryer by different frequency and different normalisation methods with electrical capacitance tomography. Powder Technol..

[4] Xiong X., Zhang Z., Liu S., Lei J. (2010). Wavelet enhanced visualization of solids distribution in the top of a CFB. Chem. Eng. J..

[5] Zhao T., Takei M., Doh D.H. (2010). ECT measurement and CFD-DEM simulation of particle distribution in a down-flow fluidized bed. Flow Meas. Instrum..

[6] Hamidipour M., Larachi F. (2010). Dynamics of filtration in monolith reactors using electrical capacitance tomography. Chem. Eng. Sci..

[7] Niedostatkiewicz M., Tejchman J., Chaniecki Z., Grudzien K. (2009). Determination of bulk solid concentration changes during granular flow in a model silo with ECT sensors. Chem. Eng. Sci..

[8] Liu S., Chen Q., Xiong X., Zhang Z., Lei J. (2008). Preliminary study on ECT imaging of flames in porous media. Meas. Sci. Technol..

[9] Makkawi Y., Ocone R. (2007). Integration of ECT measurement with hydrodynamic modelling of conventional gas-solid bubbling bed. Chem. Eng. Sci..

[10] Du B., Warsito W., Fan L.S. (2006). Imaging the choking transition in gas-solid risers using electrical capacitance tomography. Ind. Eng. Chem. Res..

[11] Xie C.G., Huang S.M., Hoyle B.S., Thorn R., Lenn C., Snowden D., Beck M.S. (1992). Electrical capacitance for flow imaging: system model for development of image reconstruction algorithms and design of primary sensors. IEE Proc. G.

[12] Tikhonov A.N., Arsenin V.Y. (1977). Solution of Ill-posed Problems.

[13] Landweber L. (1951). An iteration formula for fredholm integral equations of the first kind. Am. J. Math..

[14] Yang W.Q., Spink D.M., York T.A., McCann H. (1999). An image reconstruction algorithm based on Landweber's iteration method for electrical capacitance tomography. Meas. Sci. Technol..

[15] Jang J.D., Lee S.H., Kim K.Y., Choi B.Y. (2006). Modified iterative Landweber method in electrical capacitance tomography. Meas. Sci. Technol..

[16] Liu S., Fu L., Yang W.Q., Wang H.G., Jiang F. (2004). Prior-online iteration for image reconstruction with electrical capacitance tomography. IEE Proc. Sci. Meas. Technol..

[17] Yang W.Q., Peng L.H. (2003). Image reconstruction algorithms for electrical capacitance tomography. Meas. Sci. Technol..

[18] Mou C.H., Peng L.H., Yao D.Y., Xiao D.Y. (2005). Image reconstruction using a genetic algorithm for electrical capacitance tomography. Tsinghua Sci. Technol..

[19] Takei M. (2006). GVSPM image reconstruction for capacitance CT images of particles in a vertical pipe and comparison with the conventional method. Meas. Sci. Technol..

[20] Soleimani M., Lionheart W.R.B. (2005). Nonlinear image reconstruction for electrical capacitance tomography using experimental data. Meas. Sci. Technol..

[21] Wang H.X., Tang L., Cao Z. (2007). An image reconstruction algorithm based on total variation with adaptive mesh refinement for ECT. Flow Meas. Instrum..

[22] Fang W.F. (2004). A nonlinear image reconstruction algorithm for electrical capacitance tomography. Meas. Sci. Technol..

[23] Lei J., Liu S., Guo H.H., Li Z.H., Li J.T., Han Z.X. (2011). An image reconstruction algorithm based on the semiparametric model for electrical capacitance tomography. Comp. Math. Appl..

[24] Ortiz-Aleman C., Martin R., Gamio J.C. (2004). Reconstruction of permittivity images from capacitance tomography data by using very fast simulated annealing. Meas. Sci. Technol..

[25] Warsito W., Fan L.S. (2001). Neural network based multi-criterion optimization image reconstruction technique for imaging two-and three-phase flow systems using electrical capacitance tomography. Meas. Sci. Technol..

[26] Banasiak R., Soleimani M. (2010). Shape based reconstruction of experimental data in 3D electrical capacitance tomography. NDT & E Int..

[27] Kortschak B., Wegleiter H., Brandstatter B. (2007). Formulation of cost functionals for different measurement principles in nonlinear capacitance tomography. Meas. Sci. Technol..

[28] Lei J. (2008). Research on Image Reconstruction Algorithms of Electrical Capacitance Tomography for the Multiphase Flow. PhD thesis.

[29] Watzenig D., Brandner M., Steiner G. (2007). A particle filter approach for tomographic imaging based on different state-space representations. Meas. Sci. Technol..

[30] Soleimani M., Vauhkonen M., Yang W.Q., Peyton A., Kim B.S., Ma X.D. (2007). Dynamic imaging in electrical capacitance tomography and electromagnetic induction tomography using a Kalman filter. Meas. Sci. Technol..

[31] Soleimani M., Mitchell C.N., Banasiak R., Wajman R., Adler A. (2009). Four-dimensional electrical capacitance tomography imaging using experimental data. Prog. Electromagn. Res..

[32] Waterfall R.C., He R., White N.B., Beck C.M. (1996). Combustion imaging from electrical impedance measurements. Meas. Sci. Technol..

[33] Peng L.H., Ye J.M., Lu G., Yang W.Q. (2012). Evaluation of effect of number of electrodes in ECT sensors on image quality. IEEE Sens. J..

[34] Zhang Z.L., Huang Q.Y., Wen H.Y., Deng Y.J. (2007). Deformation Monitoring Analysis and Prediction for Engineering Constructions.

[35] Candes E.J., Li X., Ma Y., Wright J. (2011). Robust principle component analysis?. J. ACM.

[36] Wright J., Peng Y., Ma Y., Ganesh A., Rao S. Robust principal component analysis: exact recovery of corrupted low-rank matrices via convex optimization.

[37] Gao H., Cai J.F., Shen Z., Zhao H. (2011). Robust principle component analysis based four-dimensional computed tomography. Phys. Med. Biol..

[38] Goldstein T., Osher S. (2009). The split Bregman method for L1-regularized problems. SIAM J. Imag. Sci..

[39] Yin W., Osher S., Goldfarb D., Darbon J. (2008). Bregman iterative algorithms for L1 minimization with applications to compressed sensing. SIAM J. Imag. Sci..

[40] Zhang X., Burger M., Bresson X., Osher S. (2010). Bregmanized nonlocal regularization for deconvolution and sparse reconstruction. SIAM J. Imag. Sci..

[41] Beck A., Tebouule M. (2009). A fast iteration shrinkage-thresholding algorithm for linear inverse problems. SIAM J. Imag. Sci..

[42] Duchi J., Singer Y. (2009). Efficient online and batch learning using forward backward splitting. J. Mach. Learn. Res..

[43] Combettes P.L., Wajs V.R. (2005). Signal recovery by proximal forward-backward splitting. SIAM J. Multiscale Model. Simul..

[44] Montefusco L.B., Lazzaro D., Papi S., Guerrini C. (2011). A fast compressed sensing approach to 3D MR image reconstruction. IEEE Trans. Med. Imag..

[45] Cai J.F., Candes E.J., Shen Z. (2008). A singular value thresholding algorithm for matrix completion. SIAM J. Optim..

